# Looking Beyond Linear: A Closer Examination of the Relationship Between Wisdom and Wellbeing

**DOI:** 10.1007/s10902-022-00540-3

**Published:** 2022-06-18

**Authors:** Judith Glück, Nic M. Weststrate, Andreas Scherpf

**Affiliations:** 1 Department of Psychology, University of Klagenfurt, Klagenfurt, Austria; 2 Department of Educational Psychology, University of Illinois at Chicago, Chicago, USA

**Keywords:** Wisdom, Well-being, Non-linear relationships, Necessary Condition Analysis

## Abstract

There has been some controversy about the relationship between wisdom and constructs of the well-being complex. Some wisdom researchers argue that the ability to maintain a high level of well-being, even in the face of very negative experiences, is a core characteristic of wisdom. Other researchers argue that the willingness of wise people to reflect on the darker sides of life might jeopardize well-being. Studies mostly found moderate positive correlations of well-being with self-report wisdom measures and negative, zero, or low positive correlations with open-ended measures of wisdom. This paper tests the hypothesis that the relationship between wisdom and well-being is triangular rather than linear, with highly wise people being high in well-being, but people high in well-being not necessarily being highly wise. A sample of 155 participants (age 23 to 90 years) completed four wisdom measures and three measures from the well-being complex. We analyzed both linear relationships (using correlations) and triangular relationships (using Necessary Condition Analysis). Correlations of well-being with open-ended measures of wisdom were mostly insignificant; correlations with self-report measures of wisdom were mostly significant. However, scatterplots showed the expected triangular relationships and Necessary Condition Analysis indicated medium to large effect sizes for both open-ended and self-report wisdom measures. In sum, our findings show that even if wise individuals think more deeply about difficult aspects of the human existence, they are still able to maintain high levels of well-being.

## Introduction

How is wisdom related to well-being? Are very wise people happier, more satisfied with their lives; do they experience more meaning and purpose in their lives than most of us do? There has been some controversy about the relationship between wisdom and variables of the well-being complex, such as happiness, life satisfaction, and eudaimonic well-being. Modern philosophical accounts consistently emphasize that one of the key characteristics of wisdom is knowing how to live well (e.g., Grimm, 2015; Kekes, 1983; Nozick, 1989; Ryan, 2012). Although philosophers make a point of not specifying criteria for living well, one could argue that some variables of the well-being complex, such as being satisfied with one’s life, having positive and enriching relationships with others, or pursuing self-defined and worthy goals, should be characteristic of living well. In the field of wisdom psychology, some scholars, most prominently Monika Ardelt (e.g., Ardelt, 2019), have argued that the ability to maintain a high level of well-being even in the face of very negative experiences is a core characteristic of wisdom. In contrast, other researchers have argued that the willingness of wise people to reflect on the darker sides of human existence and their ability to see through positive illusions might jeopardize, not bolster, well-being (Staudinger & Glück, 2011; Staudinger & Kunzmann, 2005).

Part of this controversy can be explained by differences in how researchers conceptualize wisdom. Broadly speaking, some models of wisdom focus on wise thinking, while others focus on wise personality, values, and affect (e.g., Bauer et al., 2019; Glück & Weststrate, in press; Grossmann et al., 2020). It seems plausible that wisdom viewed as a cognitive quality—complex and multiperspectival thinking about difficult questions of life—would be less strongly and less positively related to well-being than wisdom viewed as a quality of personality, affect, and value orientations. In the following, we briefly review the most prominent conceptions and measure of wisdom from both groups and the empirical evidence concerning their relationships with measures of subjective and psychological well-being. We focus our review on “classical” measures of the well-being complex, such as subjective well-being (e.g., life satisfaction, positive and negative affect; Diener, 1984) and psychological well-being (e.g., purpose in life, personal growth, autonomy; Ryff, 1989; Ryff & Keyes, 1995). A review of relationships between wisdom and a broader range of well-being-related measures (including, for example, ego development and emotion regulation) is presented by Ardelt (2019).

Conceptions of subjective well-being focus on hedonic aspects of happiness, i.e., on pleasure and positive affect, and on more generalized appraisals of the affective quality of one’s life such as life satisfaction. Conceptions of psychological well-being take a eudaimonic approach, looking at the psychological experiences that make individual lives meaningful. It seems likely that aspects of eudaimonic well-being such as value-related purpose in life, meaningful activities, positive relationships, and an orientation towards growth would be more closely related to wisdom than hedonic aspects. Weststrate and Glück (2017b) have argued, however, that highly wise individuals might also be higher in hedonic well-being than other people in spite of, or even because of, their experience and willingness to engage with the more difficult sides of life. Arguably, as highly wise individuals are aware of the uncertainty and unpredictability of life, they have learned to appreciate and relish even small pleasures, and as they know a lot about life and about themselves and are quite independent of external reinforcement, they tend to live their lives in accordance with their personal needs and priorities.

### Models of Wisdom, Measures of Wisdom, and Relationships with Well-Being

#### Cognitive Conceptions of Wisdom

The first psychological research program on wisdom was started in the 1980s at the Max Planck Institute for Human Development in Berlin, Germany. In the Berlin Wisdom Model, Paul Baltes and colleagues defined wisdom as expert knowledge about the fundamental pragmatics of life (Baltes & Staudinger, 2000). According to these authors, wise thinking about difficult life problems is characterized by five criteria: (1) factual and (2) procedural knowledge about the issue at hand, (3) value relativism (acknowledging and accepting the fact that people have different values and goals), (4) lifespan contextualism (taking historical, cultural, and individual context into account in interpreting people’s behavior), and (5) recognition and management of uncertainty (being aware of the unpredictability of life and considering it when making decisions). To measure wisdom, Baltes and colleagues developed the Berlin Wisdom Paradigm (BWP; Staudinger et al., 1994). In a think-aloud setting, participants are presented with brief descriptions of difficult life problems; response transcripts are rated for the five criteria.

In other words, the Berlin wisdom model is a largely cognitive-oriented conception of wisdom that focuses on how individuals think about theoretical life problems. Table 1 reviews empirical findings on relationships of BWP scores with variables of the well-being complex. [Note that Mickler and Staudinger (2008) factor-analyzed the psychological well-being subscales (Ryff & Keyes, 1995) and labeled one of the two resulting factors “subjective well-being;” we still subsumed both factors under psychological well-being.] While results vary somewhat across studies, correlations of the BWP with subjective well-being are mostly small or zero. Interestingly, Kunzmann and Baltes (2003) found small negative correlations of the BWP with positive affect (*r* = − 0.17) and negative affect (*r* = − 0.13), but a positive correlation (*r* = 0.28) with affective involvement (e.g., feeling interested, attentive, or inspired). They argued that “[t]hese findings correspond to the theoretical notion that wisdom involves adherence to the reality principle (i.e., motivation to explore and understand the complexity of reality) rather than the pleasure principle (i.e., motivation to maximize pleasant experiences)” (Kunzmann & Baltes, 2003, p. 1114). Among the components of psychological well-being, personal growth had the comparatively strongest relationship with the BWP in two of the four studies; correlations with the other subscales of the Psychological Well-Being Scale are mostly around zero. Interestingly, Wink and Staudinger (2016) found by far the highest correlation (*r* = 0.55) between a growth factor of psychological well-being and the BWP. One possible explanation is that these authors studied an older sample with a narrower age range (68–77 years) than the other studies using the BWP; the link between wisdom and well-being may be stronger in old age (see, e.g., Ardelt & Edwards, 2016; Ardelt & Jeste, 2018).

**Table 1:** Correlations of Cognitive Measures of Wisdom with Measures of Subjective and Psychological Well-Being

Wisdom measure	Subjective well-being	Psychological well-being
Berlin Wisdom Paradigm (General)	Kunzmann & Baltes, (2003): Positive affect: -.17, p < .05 Negative affect: -.13, p < .05 Affective engagement: .28, p < .01 Mickler & Staudinger, (2008): Life satisfaction: .20, p < .05 Positive affect: .11, n.s. Negative affect: -.06, n.s.	Glück & Baltes, (2006): Autonomy: .09, n.s.^1^ Environmental mastery: .15, p < .01^1^ Personal growth: .30, p < .01^1^ Positive Relations to others: .12, p < .05^1^ Purpose in life: .19, p < .01^1^ Self-acceptance: .02, n.s.^1^ Glück et al., (2013): Autonomy: .19, n.s.^1^ Environmental mastery: -.02, n.s.^1^ Personal growth: .17, n.s. Positive relations to others: .02, n.s.^1^ Purpose in life: .07, n.s.^1^ Self-acceptance: .00, n.s. Mickler & Staudinger, (2008): Maturity factor (personal growth and purpose in life): .11, n.s. “Subjective Well-Being” factor (autonomy, environmental mastery, and self-acceptance): .02, n.s. Staudinger et al., (1997): Autonomy: n.s. Environmental mastery: n.s. Personal growth: .29, p < .01 Purpose in life: n.s. Relations to others: n.s. Self-acceptance: n.s. Wink & Staudinger, (2016): Personality adjustment (environmental mastery, positive relations, and self-acceptance): .24, p < .01 Personality growth (personal growth, purpose in life, and autonomy): .55, p < .01
Bremen Wisdom Paradigm (Personal)	Mickler & Staudinger, (2008): Life satisfaction: -.06, n.s. Positive affect: .09, n.s. Negative affect: .04, n.s.	Mickler & Staudinger, (2008): Maturity factor (personal growth and purpose in life): .28, p < .01 “Subjective Well-Being” factor (autonomy, environmental mastery, and self-acceptance): .05, n.s.
Wise Reasoning Vignettes	Grossmann et al., (2013): Life satisfaction: .17, p < .05 Positive affect: .01, n.s. Negative affect: -.27, p < .01 Depressive brooding: -.33, p < .01	
Situated Wise Reasoning Scale	Grossmann et al., (2016): Positive affect (intraindividual): B = .43 (SE_B_ = .15), p < .01 Positive affect (interindividual): B = .17 (SE_B_ = .40), n.s. Negative affect (intraindividual): B = .23 (SE_B_ = .14), n.s Negative affect (interindividual): B = -.04 (SE_B_ = .10), n.s.	

^1^ Previously unpublished data. n.s. = not significant

Ursula Staudinger and colleagues have distinguished general wisdom (i.e., the wisdom people have about people and life in general) from personal wisdom (i.e., the wisdom people have about themselves and their own life; Staudinger, 2019; Staudinger et al., 2005). They proposed a model of personal wisdom that consists of five components parallel to the Berlin model: self-knowledge (knowing one’s own strengths and weaknesses, feelings, and motives), heuristics for growth and self-regulation (having a repertoire of strategies for dealing with and learning from difficult situations), self-relativism (being willing and able to take an “outside perspective” on oneself), interrelating the self (being aware of the causes of one’s own feelings and behaviour and one’s relatedness to other people), and tolerance of ambiguity (recognizing, accepting, and managing the uncertainties and uncontrollabilities in one’s life). Thus, while this model of personal wisdom involves some non-cognitive, especially emotional aspects, it still has a clear focus on how people *think* about these aspects. To measure personal wisdom, Mickler and Staudinger (2008) proposed an interview-based measure that focuses on participants’ strengths and weaknesses as a friend. As in the BWP, responses are rated with respect to the five criteria. As Table 1 shows, Mickler and Staudinger (2008) found zero correlations of this measure of personal wisdom with indicators of both subjective and psychological well-being.

Igor Grossmann and colleagues (e.g., Grossmann, 2017; Grossmann et al., 2020) have conducted a highly productive research program on wise reasoning, which is characterized by metacognitive capacities such as epistemic humility, considering and balancing multiple perspectives, and adapting to different contexts (Grossmann et al., 2020). Importantly, Grossmann and colleagues conceive of wisdom as situationally variable rather than as a stable trait; they have demonstrated that wise reasoning shows considerable intraindividual variability (Grossmann, 2017; Grossmann et al., 2016). To measure wise reasoning, Grossmann and colleagues have developed vignette-based open-ended measures (e.g., Grossmann et al., 2013) as well as the Situational Wise Reasoning Scale (SWIS), a self-report scale that assesses wise reasoning with respect to a specific past situation (Brienza et al., 2018). For a vignette-based measure, Grossmann et al. (2013) reported a correlation of *r* = 0.17 with life satisfaction, and, interestingly, somewhat higher negative correlations with negative affect and depressive brooding. Using the SWIS, Grossmann et al. (2016) found that thinking more wisely was correlated intraindividually (but not interindividually) with more intense positive emotions.

In sum, measures that focus on wise thinking tend to have zero or relatively small correlations with variables of the well-being complex—with the possible exception of personal growth, a subscale of the psychological well-being scale that looks at whether individuals see themselves as constantly developing and view new experiences as opportunities for growth and learning. According to the correlations reviewed in Table 1, individuals who are able to think about complex life problems in ways that are considered wise—being aware of the limitations of one’s own knowledge, the multiplicity of possible perspectives and values, and the importance of context—do not generally seem to be much happier, more satisfied with their lives, or higher in psychological components of well-being than individuals who are less able to think wisely, although one study suggests that the relationship between cognitive aspects of wisdom and growth-related aspects of well-being may be stronger in older adults (Wink & Staudinger, 2016). Intraindividually, wise reasoning may accompany elevated positive affect, although the direction of this relationship is unclear. Alternatively, people might be better able to reason wisely when they are in a more positive emotional state (Glück & Weststrate, in press).

As mentioned earlier, researchers from the Berlin group have argued that cognitively wiser individuals may engage more deeply with the darker sides of our existence than less wise individuals, which might even suggest a negative relationship between wisdom and well-being (Kunzmann & Baltes, 2003; Staudinger & Glück, 2011; Staudinger & Kunzmann, 2005). The evidence for that claim, however, comes from only one study (Kunzmann & Baltes, 2003) that only looked at relationships between wisdom and affect.

#### Non-Cognitive Conceptions of Wisdom

Since the early 2000s, a growing number of psychological wisdom conceptions have been developed that focus less on wise thinking and more on wise personality, motivational, and affective characteristics, i.e., on how wise people experience life and its challenges (Glück & Weststrate, 2021). Most prominently, Monika Ardelt (2003, 2004) argued that wisdom is more than a way of thinking because it is deeply rooted in personal emotional experiences. Ardelt (2003) defined wisdom as a combination of three personality dimensions. The cognitive dimension describes a desire for a deep understanding of life and other people. The reflective dimension describes a willingness to look at issues from different perspectives, include looking at one’s own behavior from the perspective of others. The affective or, in more recent publications, compassionate dimension describes a deep concern for other people and humanity at large. As mentioned earlier, Ardelt considers a high level of well-being as a core characteristic of highly wise individuals; she has argued that “[W]ise individuals have gained the equanimity to preserve subjective well-being and inner peace even when confronted with hardship and adversity in life” (Ardelt, 2019, p. 606). Ardelt (2003) developed the Three-Dimensional Wisdom Scale (3DWS), a 39-item self-report scale that assesses the three personality dimensions. Using the 3DWS, Ardelt and colleagues have conducted the largest research program to date on relationships between wisdom and well-being. As Table 2 shows, they have quite consistently found relatively strong relationships, with far more correlations above *r* = 0.40 than for any of the cognitive wisdom measures.

**Table 2:** Correlations of Non-Cognitive Measures of Wisdom with Subjective and Psychological Well-Being

Wisdom measure	Subjective well-being	Psychological well-being
Adult Self-Transcendence Inventory	Le (2011): Life satisfaction: .09, n.s. Beaumont (2009): Subjective happiness: .48, p < .01	Glück et al. (2013): Autonomy: .31, p < .01^1^ Environmental mastery: .31, p < .01^1^ Personal growth: .22, p < .01 Purpose in life: -.07, n.s.^1^ Positive relations to others: .31, p < .01^1^ Self-acceptance: .33, p < .01 Levenson et al. (2005): Alienation: -.25, p < .01 Beaumont (2009): Meaning in life: .40, p < .01 Self-actualization: .48, p < .01
San Diego Wisdom Scale	Thomas et al. (2019): Depressive symptoms: -.08 [-.17, .01] Life satisfaction:.14 [.05, .23] Happiness: .13 [.04, .21] Jeste et al. (2021): Depressive symptoms: -.49, p < .01 Happiness: .54, p < .01	
Self-Assessed Wisdom Scale	Webster et al. (2014): Hedonistic mental health: .30, p < .01 Hayat et al. (2016): Life satisfaction (sample 1): .18, p < .05 Life satisfaction (sample 2): .31, p < .01 Webster and Deng (2015): Self-esteem: .20, p < .01 Webster et al. (2018): Self-esteem: .26, p < .01	Ardelt (2011): Autonomy: .32, p < .01 Environmental mastery: .17, p < .05 Personal growth: .51, p < .01 Purpose in life: .24, p < .01 Positive relations to others: .34, p < .01 Self-acceptance: .43, p < .01 Glück et al. (2013): Autonomy: .24, p < .01 ^1^ Environmental mastery: .15, p < .05 ^1^ Personal growth: .28, p < .01 Purpose in life: .04, n.s.^1^ Positive relations to others: .23, p < .01^1^ Self-acceptance: .17, p < .01 Webster et al. (2014): Eudaimonic mental health: .44, p < .01 Webster (2003): Ego integrity: .23, p < .05 Webster (2010): Ego integrity: .45, p < .01 Purpose in life: .35, p < .01 Webster et al. (2018): Meaning in life: .33, p < .01 Webster and Deng (2015): Ego integrity: .33, p < .01 Taylor et al. (2011): Psychological well-being: .46, p < . 01
Three-Dimensional Wisdom Scale	Ardelt (2003): General well-being: .45, p < .01 Depressive symptoms: -.59, p < .01 Ardelt and Edwards (2016)^2^: Subjective well-being: .49, p < .01 Etezadi and Pushkar (2013): Positive affect: r = .34, p < .01 Negative affect: r = -.27, p < .01 Le (2011): Life satisfaction: .33, p < .01 Zacher et al. (2013): Life satisfaction (sample 1): .35, p < .01 Positive affect (sample 1): .33, p < .01 Negative affect (sample 1): -.40, p < .01 Life satisfaction (sample 2): .16, p < .01 Positive affect (sample 2): .14, p < .01 Negative affect (sample 2): -.29, p < .01 Cheung and Chow (2020): Personal well-being: .66, p < .01 Thomas et al. (2017): Depressive symptoms: r = -.32 [-.37, -.22] Life satisfaction: r = .30 [.26, .35] Happiness: r = .35 [.31, .39]	Ardelt (2003): Purpose in life: .61, p < .01 Ardelt (2011): Autonomy: r = .41, p < .01 Environmental mastery: .40, p < .01 Personal growth: r = .52, p < .01 Purpose in life: r = .45, p < .01 Positive relations to others: .48, p < .01 Self-acceptance: .49, p < .01 Ardelt and Edwards (2016)^2^: Purpose in life: .34, p < .01 Glück et al. (2013): Autonomy: .17, p < .05^1^ Environmental mastery: .34, p < .01^1^ Personal growth: .41, p < .01 Purpose in life: .17, p < .05^1^ Positive relations to others: .46, p < .01^1^ Self-acceptance: .37, p < .01 Taylor et al. (2011): Psychological well-being: .64, p < . 01 Etezadi and Pushkar (2013): Meaning in life: .35, p < .01 Mansfield et al. (2010): Psychological well-being: -.13, n.s. Ardelt and Ferrari (2019): Purpose in life (sample 1): .25, p < .05 Purpose in life (sample 2): .51, p < .01

^1^ Previously unpublished data. n.s. = not significant

^2^ Reported in Ardelt (2019); Ardelt and Edwards (2016) did not report zero-order correlations

Jeffrey Dean Webster’s conceptualization of wisdom focuses on people’s willingness to gain insights from life experiences and to apply those insights for the good of themselves and others (Webster, 2003, 2007). According to Webster, wisdom is characterized by life experience, a willingness to reminisce and reflect on experiences, openness to experiences and ideas, emotional regulation, and humor. The Self-Assessed Wisdom Scale (SAWS; Webster, 2003, 2007), measures these five components of wisdom. As Table 2 shows, correlations with well-being tend to be somewhat lower than for the 3DWS, but SAWS scores are clearly positively related to well-being.

Michael R. Levenson and colleagues (2005; Aldwin et al., 2019) have proposed a conception of wisdom that is quite closely related to well-being. They argue that the core characteristic of wisdom is self-transcendence: accepting oneself as one is, overcoming one’s ego, being independent of external sources of self-esteem such as power or fame, and feeling connected to others and the world at large. The Adult Self-Transcendence Inventory (ASTI; Levenson et al., 2005; for a more recent version see Koller et al., 2017) is a self-report scale that measures wisdom as self-transcendence. Interestingly, in spite of its conceptual overlap with well-being, correlations of the ASTI with well-being do not seem to be higher than those of other self-report measures of wisdom.

The San Diego Wisdom Scale (SD-WISE; Thomas et al., 2019) measures six components of wisdom that are assumed to have a neurobiological basis (Meeks & Jeste, 2009): social advising, decisiveness, emotional regulation, insight, pro-social behaviors, and tolerance for divergent values. As Table 2 shows, correlations of the SD-WISE with aspects of well-being differ markedly across the two available studies, with, for example, an insignificant correlation with depressive symptoms found by Thomas et al. (2019) and a correlation of − 0.49 found by Jeste et al. (2021).

In sum, Table 2 suggests that the correlations of non-cognitive wisdom measures with variables of the well-being complex are generally significant and higher than those of cognitive wisdom measures, but there is considerable variability between studies. Correlations with subjective well-being are typically in the 0.30 to 0.50 range for the 3DWS; the other wisdom measures show more variability. Correlations with psychological well-being (PWB) vary considerably between studies and between PWB subscales, with relatively high correlations for personal growth and somewhat more variability for the other subscales.

In sum, some recent conceptions of wisdom put less focus on cognitive aspects, such as knowledge and expertise, and more focus on non-cognitive aspects, such as openness, compassion, emotion regulation, humor, or self-transcendence. Conceptually, it makes sense that those qualities would be more closely related to well-being than wisdom-related ways of thinking, and as Table 2 shows, these measures indeed tend to show higher correlations with well-being.

These findings may bring up the question whether cognitive and non-cognitive measures of wisdom even assess the same basic construct. Recently, Judith Glück and colleagues have aimed to integrate cognitive and non-cognitive models of wisdom (see also Bauer et al., 2019, for a different account of wisdom as a combination of complex thinking and an eudaimonic orientation). Glück and Weststrate (in press) have argued that in challenging real-life situations, non-cognitive components of wisdom such as emotion regulation, openness, and a concern for a common good are necessary for individuals to be able to access their wisdom-related cognitive capacities. In other words, even if cognitive and non-cognitive components of wisdom are not strongly related in population samples, they may come together in particularly wise individuals. The MORE Life Experience Model (Glück & Bluck, 2013; Glück et al., 2019) is a theory of the development of wisdom that proposes that wisdom-related knowledge and reasoning capacities develop as people reflect on challenging life experiences; non-cognitive resources such as openness, reflectivity, or empathy are necessary for gaining wisdom-fostering insights from such experiences. The MORE resources are measured using interviews about difficult autobiographical experiences (Glück et al., 2019). Relationships between the MORE wisdom resources and variables of the well-being complex will be reported in this paper.

#### The Potential Role of Method Variance

The considerable difference in size of correlations between Tables 1 and 2 is likely to reflect the substantive difference between conceptions of wisdom as mostly a cognitive capacity and conceptions of wisdom as mostly a non-cognitive quality involving personality, motivational, or affective characteristics. As discussed earlier, if wisdom is largely a way of thinking about life and its challenges, wisdom might be unrelated or perhaps, in some situations, even negatively related to well-being. If wisdom is largely a characteristic of personality and affect that leads to an open-minded, caring, and emotionally balanced outlook on life, as non-cognitive conceptions of wisdom suggest, then it should be substantially positively correlated with well-being.

It is important to note, however, that part of the differences in correlations in Tables 1 and 2 may also be caused by method variance (Glück, 2018). The non-cognitive measures of wisdom in Table 2 are all self-report scales (overview in Webster, 2019), as are the variables of the well-being complex. Most self-report scales typically assess people’s broad, decontextualized views of themselves, as do the typical well-being measures. In our experience, self-report scales that measure positively valued constructs are almost always correlated, with the common variance representing a general positivity of individuals’ self-images. Also, self-report measures of both well-being and wisdom tend to have somewhat skewed distributions, with few people scoring below the respective scale midpoints. Some researchers have argued that that if wisdom involves self-reflection and self-criticism, then, paradoxically, individuals may actually describe themselves less positively in self-report scales than less wise people do (Aldwin, 2009; Glück, 2018).

On the other hand, all cognitive wisdom measures listed in Table 1 except the Situated Wise Reasoning Scale are open-ended measures where people are presented with a wisdom-requiring problem or question and asked to produce a response; responses are then scored for pre-defined wisdom criteria (overview in Kunzmann, 2019). That is, these measures do not assess people’s views of themselves but the way people actually think (and talk or write) about specific life problems. Open-ended wisdom measures tend to be more difficult than self-report scales, with relatively few people scoring above the scale midpoint. In other words, non-cognitive wisdom measures differ from most cognitive wisdom measures in response format (Likert-type scales vs. open-ended written or spoken responses), specificity (general self-descriptions vs. responses to specific problems), and “difficulty” (high percentage vs. low percentage of high scores). All these method factors may influence the relationship between wisdom and other variables and would suggest stronger relationships of well-being with non-cognitive wisdom measures (self-report scales) than with cognitive wisdom measures (open-ended measures) even if the wisdom models underlying both types of measures were the same.

This paper investigates the role of both content and method variance by analyzing the relationships between four measures of wisdom—two open-ended measures and two self-report measures (one of each with more cognitive content, the other with more non-cognitive content)—and three measures representing different facets of the well-being complex. We expected to find stronger relationships between wisdom and well-being for non-cognitive compared to cognitive measures (and subdimensions of measures) and for self-report measures compared to open-ended measures of wisdom. In addition, however, we believe that correlations may not be the most conceptually adequate way to analyze the relationship between wisdom and well-being, as we discuss next.

### The Assumption of Linear Relationships Between Wisdom and Well-Being

To date, all reported evidence on the relationship between wisdom and variables of the well-being complex has been correlational. However, correlations test for a linear relationship between two variables, which may not be the best representation of the theoretical relationship between wisdom and well-being. Most wisdom researchers would probably agree that there are far more people high in well-being than people high in wisdom. As Bauer et al. (2019) put it, “The mere presence of wellbeing—even eudaimonic well-being—is not grounds for the assessment of wisdom” (p. 93). For example, Staudinger and Kunzmann (2005) distinguished between two developmental pathways. People on the “adjustment path” aim to live a happy, satisfied life without asking too many in-depth questions about its meaning or purpose. People on the “growth path” are driven by a deep desire to understand and learn, which leads them to look deeply into the meaning of sad or scary experiences and to critically question themselves. Highly wise individuals, according to Staudinger and Kunzmann, are high in both adjustment and growth. Weststrate and Glück (2017a) looked at a similar distinction in the ways study participants reflected about difficult life events. Redemptive reflection involved finding a positive ending to the story and was correlated with well-being, whereas exploratory reflection involved gaining insights from the experience and was correlated with wisdom (see also Bauer et al., 2019; King et al., 2000; Pals, 2006).

In other words, there are easier paths to happiness and well-being than in-depth reflection and growth. It may be a lot easier to think about challenging events in ways that maintain or reinforce one’s self-esteem, without digging too deep into the scary or sad implications of an experience. These ideas suggest that even if wisdom involves a high level of happiness, there should be far more happy people than wise people. At the same time, it seems somewhat unlikely that very wise individuals would be very unhappy. Wisdom has been related to variables like gratitude (König & Glück, 2014) and forgiveness (Taylor et al., 2011); also, the idea that wise individuals are deeply at peace with themselves and the world is part of folk conceptions of wisdom (overview in Weststrate et al., 2019). In a theoretical chapter, Weststrate and Glück (2017b) attempted to reconcile the different perspectives and inconsistent findings by arguing that the developmental pathway to wisdom requires confrontation with negative experiences and feelings, but having reached a high level of wisdom involves being at peace with oneself, one’s life, and the world. Specifically, they argued that there are three reasons why wise individuals should be high in well-being: (1) wise individuals have learned from experience to handle difficult life situations well; (2) wise individuals appreciate even small positive moments because they are acutely aware of the less pleasant sides of life; and (3) wise individuals know themselves and what they need to live a good life, and they design their lives in ways that fulfill those needs. In addition, even if highly wise individuals are more likely to look into the difficult and negative sides of life than other people—for example, through occupational choices and volunteering activities, through a willingness to reflect on their own weaknesses and mistakes, or through increased awareness of the uncertainty and finitude of life—these more fleeting psychological states might not manifest themselves in typical well-being measures, which assess people’s summative, overall evaluations of their life and personal values and traits.

**Fig. 1:**
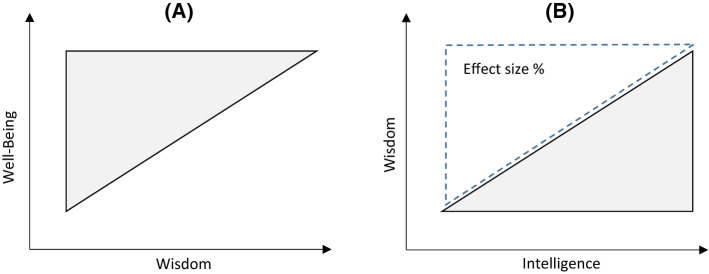
Triangular Relationships Between **a** Wisdom and Well-being and **b** Intelligence and Wisdom

In sum, these considerations suggest that the assumption of linearity underlying correlational analyses may not be a good representation of how wisdom and variables of the well-being complex are related. If highly wise individuals are, indeed, high in well-being, but there are far more people high in well-being than in wisdom, then the relationship between the two variables should be triangular rather than linear, as shown in Fig. 1, Panel (A). People high in wisdom should be high in well-being; people low in wisdom may also be high in well-being or low in well-being or anywhere in between. In fact, as the results of the current study will show, even if the correlation between two variables is small or zero there can still be a triangular relationship between the two variables.

**Fig. 2:**
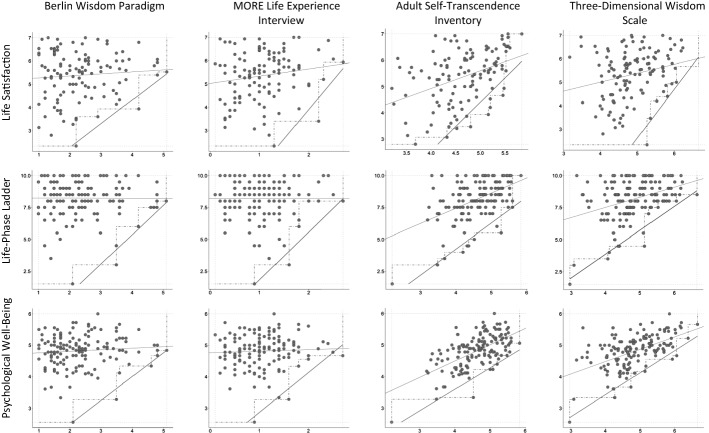
Scatterplots of Wisdom and Well-Being Scores Depicting Non-Linear Associations

This paper analyzes linear as well as triangular relationships between wisdom and well-being. Fortunately for us, Jan Dul (2016) recently developed Necessary Condition Analysis (NCA), a method for analyzing triangular relationships where one variable is a necessary but not sufficient condition for another variable^1^. If, for example, intelligence were necessary but not sufficient for wisdom, all wise individuals would be relatively intelligent, but not all intelligent individuals would be wise. Panel (B) in Fig. 1 illustrates this type of relationship. Dul (2016, 2020) suggested to use the relative size of the empty area in the top left of the scatterplot as the effect size for a necessary-condition relationship. The relationship between wisdom and well-being cannot easily be described in terms of necessary conditions from a theoretical perspective, but technically, it is of the same nature. If all highly wise individuals are high in well-being, but not all people high in well-being are highly wise, then high well-being is technically a necessary but not sufficient condition for high wisdom. Therefore, we used the computational methods developed by Dul (2016) for NCA to estimate effect sizes for a triangular relationship between wisdom and well-being. As our theoretical considerations would suggest that wisdom is more likely to lead to well-being than vice versa, the scatterplots we show in Fig. 2 display wisdom on the x axis and well-being on the y axis. For the statistical analyses, however, we flipped the scatterplots so that we could use Dul’s (2020) R routines to estimate the relative size of the empty area on the bottom right side of plots like Panel (A) in Fig. 1.

We believe that analyzing triangular relationships between wisdom and well-being may be particularly interesting with respect to open-ended measures of wisdom. As mentioned earlier, measures like the Berlin Wisdom Paradigm tend to be relatively “difficult,” with many participants scoring low and few participants scoring relatively high, whereas in well-being measures, the large majority of participants tend to score above the scale midpoint. Therefore, even if high wisdom involves high well-being, there still has to be a large number of participants high in well-being and low in wisdom, which might cause a low or zero correlation. If that were the case, it would suggest that even the cognitive conceptions of wisdom that are typically assessed using open-ended measures capture more than “just” wise thinking—as Glück and Weststrate (in press) suggested, wise thinking might be reflective of a broader attitude towards life that involves non-cognitive as well as cognitive components.

### Predictions

This paper analyzes the relationships between four different measures of wisdom and three different measures of the well-being complex. As explained above, we will present scatterplots as well as correlations and effect sizes for triangular relationships. Our first prediction was that relationships would be triangular rather than linear at least for open-ended measures of wisdom (because few people score high in these measures, but many score high in well-being measures), but possibly for all measures of wisdom (because there are many ways to happiness and, therefore, more highly happy people than highly wise people).

Second, we expected to find stronger relationships with well-being for non-cognitive measures than for cognitive measures (and subdimensions) of wisdom, because non-cognitive measures are conceptually closer to well-being as they involve aspects of personality, values, and affect. Some non-cognitive wisdom measures, such as the ASTI, explicitly include aspects of well-being such as self-acceptance and peace of mind (Koller et al., 2017). We also expected to find closer relationships with well-being for self-report measures than for open-ended measures of wisdom due to shared method variance. For distinguishing between these two predictions, results for the non-cognitive subcomponents of the open-ended measures (i.e., the openness, empathy, and emotion regulation subcomponents of the MORE Life Experience Interview) and cognitive subcomponents of the self-report measures (the cognitive and reflective dimension of the 3DWS) will be particularly interesting.

Third, we expected to find some differences between the well-being measures in their relationships with wisdom. As discussed earlier, we expected wisdom to be most closely related to psychological well-being (Ryff & Keyes, 1995), a eudaimonic conception of well-being that includes six facets of living a good and meaningful life. We also expected a relatively strong relationship between wisdom and general life satisfaction (Pavot et al., 1998), an overall evaluation of participants’ past, current, and future life. Assuming that highly wise individuals indeed know how to live well, we would expect them to generally have a positive, unembittered view of their life (Glück, 2011). We expected the weakest relationship for wisdom with our adapted version of the “life phase ladder,” a measure that asked participants to compare their current life phase to the best and worse phases of their life (Cantril, 1965). We did not expect highly wise individuals to judge their current life phase as the best more frequently than less wise individuals, because wiser individuals might be more willing to aim for objectivity in evaluating their current life situation. Considering one’s current life phase as the best may be a form of self-deception or positivity bias to which highly wise individuals might be less susceptible than other people.

## Method

The data for this study were collected during the initial wave of data collection for a longitudinal study investigating the influence of life events and personal resources on the development of wisdom. A total of 155 participants filled out questionnaires and participated in two interviews of about 90 min each.

### Participants

Most participants were recruited through a random address sample obtained from the Austrian census bureau. Invitation letters and a response envelope were sent out to 3,000 people, of whom 219 (7.3%) responded and 116 (3.9%) eventually participated. Thus, while the sample was largely representative of the Austrian population in terms of gender, age, and education, the participants were probably more interested in reflecting on life experiences than most people. (This may, however, be true for many studies of wisdom.) To increase the likelihood of including relatively wise participants, 14 wisdom nominees were recruited through media calls and 25 participants were re-recruited from an earlier study (Glück et al., 2013; Weststrate & Glück, 2017a). In total, the final sample included 155 participants (85 women and 70 men) between 23 and 90 years of age (*M* = 56.2; *SD* = 14.6). In terms of education, 42.2% of the participants had completed at least the 9 years of education mandatory in Austria; 25.2% had completed the 12 school years necessary for access to university, and 32.5% had a college or university degree. Participants were paid € 100 for participation in the study.

### Measures

#### Wisdom Measures

**Self-Report Wisdom Measures** As explained earlier, we used both self-report and open-ended measures of wisdom. The two self-report scales were the Adult Self-Transcendence Inventory and the Three-Dimensional Wisdom Scale. The *Adult Self-Transcendence Inventory* (ASTI; Koller et al., 2017; Levenson et al., 2005) is a 35-item scale that defines wisdom as self-transcendence developed through stages of self-knowledge, detachment from external sources of self, and integration of all self-aspects. Sample items include, “I can learn a lot from others,” “My peace of mind is not easily upset,” and “I feel that my individual life is part of a greater whole.” The items were presented with a six-point response scale ranging from “do not agree at all” to “agree completely.” Cronbach’s alpha for the ASTI was 0.83.

The *Three-Dimensional Wisdom Scale* (3D-WS; Ardelt, 2003) measures three personality components of wisdom. The reflective dimension represents a willingness to take different perspectives on both phenomena and oneself (e.g., “Things often go wrong for me by no fault of my own;” reverse-scored). The cognitive dimension measures the desire to gain a deep understanding of issues concerning human existence (e.g., “Ignorance is bliss;” reverse-scored). The compassionate dimension describes a positive, caring attitude toward others (e.g., “Sometimes I feel a real compassion for everyone”). The 3D-WS has two parts with different response scales; in the current study we used seven-point scales (part 1: 1 = “disagree completely”, 7 = “agree completely”; part 2: 1 = “not true of myself”, 7 = “definitely true of myself”). Cronbach’s alpha for the 3D-WS was 0.86.

**Open-ended Wisdom Measures** Open-ended measures included the life review problem from the Berlin Wisdom Paradigm and the MORE Life Experience Interview. The *life review problem* was administered following the manual for the Berlin Wisdom Paradigm (BWP; Staudinger et al., 1994). Participants were first presented with some introductory tasks to get acquainted with the think-aloud procedure. Then, they were asked to think aloud about the following problem (Glück & Baltes, 2006; Staudinger & Baltes, 1996): “In reflecting over their lives, people sometimes realize that they have not achieved what they had once wanted to achieve. What could one/these persons consider and do in such a situation?” Participants’ responses were recorded and transcribed. Transcripts were evaluated by trained student raters according to the five criteria of the Berlin Wisdom Model (overview in Baltes & Staudinger, 2000), with two raters for each criterion: factual knowledge (ICC = 0.71), procedural knowledge (ICC = 0.37), value relativism (ICC = 0.60), lifespan contextualism (ICC = 0.74), and recognition and management of uncertainty (ICC = 0.69); Cronbach’s alpha for the total BWP score was 0.85.

In the *MORE Life Experience Interview* (Glück et al., 2019), participants were first asked to make a list of the most difficult challenges in their life. Then, they were interviewed about the most difficult challenge they were open to discussing. They first provided an open-ended narrative of the event and were then asked questions concerning their feelings, thoughts, and possible insights gained from the event. Responses were recoded and transcribed, and the transcripts were scored by trained student raters for the five wisdom resources of the MORE Life Experience Model (Glück & Bluck, 2013), with two raters for each criterion: mastery (ICC = 0.84), openness (ICC = 0.78), reflectivity (ICC = 0.55), emotion regulation (ICC = 0.58), and empathy (ICC = 0.62). Cronbach’s alpha for the total MORE score was 0.82.

For the purpose of the current analyses, these four measures enabled us to disentangle, to some extent, effects of content and method on the relationship between wisdom and well-being. The Berlin Wisdom Paradigm is an open-ended measure of cognitive components of wisdom. The MORE Life Experience Interview is an open-ended measure of mostly non-cognitive components of wisdom; we considered reflectivity as a cognitive component and the others as non-cognitive (Glück & Weststrate, in press). The 3DWS is a self-report measure that includes two components that we considered as cognitive: the cognitive and the reflective dimension. The ASTI is a self-report measure of a (mostly) non-cognitive conception of wisdom.

#### Measures of the Well-Being Complex

Participants were presented with three measures of different facets of the well-being complex. The *Temporal Satisfaction with Life Scale* (TSWLS; Pavot, 2014; Pavot et al., 1998) is a 15-item scale that assesses overall life satisfaction in a temporal perspective: participants respond to the same five items with respect to their part, present, and future life satisfaction. A sample item is “I am satisfied with my current life” (present), “I am satisfied with my life in the past,” and “I will be satisfied with my life in the future” (future). Participants indicate their responses on a seven-point scale (1 = strongly disagree, 7 = strongly agree). As the TSWLS was added later in the study, it was completed by only 120 participants. A total life satisfaction score was computed as the mean across the 15 items; Cronbach’s alpha was 0.91.

The second measure was a modified version of the Cantril Self-Anchoring Striving Scale (often called the “Cantril ladder;” Cantril, 1965; see also Glatzer & Gulyas, 2014). In the original Cantril ladder, participants are presented with a depiction of an 11-step ladder where the top (10) represents the best possible life they can imagine for themselves and the bottom (0) represents the worst possible life, and asked to locate the point on the ladder that best describes their current life. In the current study, the Cantril ladder was modified to represent a life-phase perspective. Participants were presented with a picture of a 10-step ladder where the top rung (10) was labeled “best time of your life” and the bottom rung (1) was labeled “worst time of your life.” They were asked to mark the rung that best represented the current phase of their life. Thus, the judgment was made relative to best and worst life phases experienced so far, rather than to best and worst possible lives. Some participants misunderstood the instructions and made their cross in the space between two rungs. These responses were coded as the number of the lower rung + 0.5. As the “life-phase ladder” is a one-item measure, internal consistency cannot be evaluated.

The third measure was an 18-item short version of the Psychological Well-Being Scale (Ryff & Keyes, 1995) that was used in several previous wisdom studies (e.g., Glück & Baltes, 2006; Glück et al., 2013). It measures six dimensions of psychological well-being using three items each: *autonomy* (e.g., “I have confidence in my opinions, even if they are contrary to the general consensus”); *environmental mastery* (e.g., “In general, I feel I am in charge of the situation in which I live”); *personal growth* (e.g., “I think it is important to have new experiences that challenge how you think about yourself and the world”); *positive relations with others* (e.g., “People would describe me as a giving person, willing to share my time with others”); *purpose in life* (e.g., “Some people wander aimlessly through life, but I am not one of them”); and *self-acceptance* (e.g., “When I look at the story of my life, I am pleased with how things have turned out”).). Participants indicated their responses on a six-point scale (1 = not at all true, 6 = completely true). A total score was computed as the average across all 18 items; Cronbach’s alpha was 0.75.

## Results

Table 3 shows descriptive statistics for all measures. As the table shows, the means for both open-ended measures of wisdom were below the scale midpoints (BWP: *M* = 2.33 on a 1–7 scale; MORE: *M* = 1.05 on a 0–3 scale), whereas the means for both self-report measures were above the scale midpoints (3DWS: *M* = 4.85 on a 1–7 scale; ASTI: *M* = 4.62 on a 1–6 scale). The means for the three well-being measures were also substantially above the scale midpoints (General Life Satisfaction: *M* = 5.34 on a 1–7 scale; Life-Phase Ladder: *M* = 8.16 on a 1–10 scale; Psychological Well-Being: *M* = 4.81 on a 1–6 scale).

**Table 3:** Descriptive Statistics for Wisdom and Well-Being Measures

	Mean	SD	Minimum–Maximum
*Wisdom measures*			
Berlin Wisdom Paradigm: Total Score	2.33	.96	1.0–5.1
Factual Knowledge	2.92	1.36	1.0–7.0
Procedural Knowledge	1.80	.80	1.0–4.5
Value Relativism	1.66	.96	1.0–5.5
Lifespan Contextualism	2.69	1.52	1.0–7.0
Uncertainty	2.59	1.51	1.0–7.0
MORE Life Experience Interview: Total Score	1.05	.53	0.1–2.7
Sense of Mastery	1.62	.93	0.0–3.0
Openness	1.24	.87	0.0–3.0
Reflectivity	1.09	.82	0.0–3.0
Empathy	.65	.63	0.0–3.0
Emotion Regulation	.66	.65	0.0–2.5
Three-Dimensional Wisdom Scale: Total Score	4.85	.72	3.0–6.7
Cognitive Dimension	4.77	.94	2.6–6.5
Reflective Dimension	5.14	.88	2.2–6.9
Compassionate Dimension	4.62	.91	2.1–6.7
Adult Self-Transcendence Inventory: Total Score	4.62	.62	2.2–5.8
*Well-being measures*			
General Life Satisfaction	5.34	1.05	2.3–7.0
Well-Being Life-Phase Ladder	8.16	1.54	1.5–10.0
Psychological Well-Being	4.81	.53	2.6–6.0

We first computed Pearson correlations between the wisdom and well-being measures, expecting to replicate earlier findings. Next, we inspected scatterplots of these relationships and performed NCA analyses computing the relative size of the empty area in each of the respective scatterplots. Dul (2016) suggested to compute the size of the empty area in the top-left corner of the scatterplot as a proportion of the entire rectangle that contains observations (cf. Panel [B] in Fig. 1). As explained earlier, we flipped each scatterplot so that the empty area that was originally in the top-left corner of each scatterplot was depicted in the bottom-right corner (with wisdom as the horizontal and well-being as the vertical axis, as in Panel [A] of Fig. 1). Dul (2016) discussed several different methods for computing the effect sizes, using different ways of drawing “ceiling lines” between the empty area and the non-empty area. He recommended CE-FDH (ceiling envelopment with free disposal hull), a line that consists of piecewise linear functions, as the default method for drawing ceiling lines. As a second option, he suggested CR-FDH (ceiling regression with free disposal hull), which “draws an OLS regression line through the upper-left edges of the CE-FDH piecewise linear function” (Dul, 2016, p. 27). Effect sizes below 0.1 are considered as small effects, effect sizes between 0.1 and 0.3 as medium effects, effect sizes between 0.3 and 0.5 as large effects, and effect sizes above 0.5 as very large effects (Dul, 2016, p. 30). Dul (2020) developed an R package for computing these indices, which we used for our analyses. Table 4 presents Pearson correlations and NCA results for the relationships between the wisdom and well-being measures; Fig. 2 shows scatterplots of the relationships.

**Table 4:** Pearson correlations between the wisdom measures and the measures of the well-being complex

Wisdom		Well-Being		
	Cognitive /Non-cognitive Focus	General Life Satisfaction	Life-Phase Ladder	Psychological Well-Being
***Open-ended measures***				
Berlin Wisdom Paradigm: Total Score	*Cognitive*	*r* = .081*, p* = .385 NCA^1^: .30/.25	*r* = -.006*, p* = .939 NCA: .31/.25	*r* = .089*, p* = .289 NCA: .29/.24
Factual Knowledge	*Cognitive*	*r* = .076*, p* = .419 NCA: .25/.21	*r* = -.055*, p* = .516 NCA: .22/.17	*r* = .033*, p* = .695 NCA: .25/.16
Procedural Knowledge	*Cognitive*	*r* = .104*, p* = .265 NCA: .34/.28	*r* = .125*, p* = .137 NCA: .49/.41	*r* = .230*, p* = .006 NCA: .46/.38
Value Relativism	*Cognitive*	*r* = .000*, p* = .996 NCA: .41/.36	*r* = .024*, p* = .774 NCA: .57/.46	*r* = .100*, p* = .235 NCA: .43/.34
Lifespan Contextualism	*Cognitive*	*r* = .117*, p* = .210 NCA: .40/.33	*r* = .040*, p* = .635 NCA: .43/.34	*r* = .082*, p* = .327 NCA: .40/.34
Uncertainty	*Cognitive*	*r* = .018*, p* = .845 NCA: .33/.28	*r* = -.092*, p* = .272 NCA: .22/.18	*r* = -.018*, p* = .831 NCA: .21/.15
MORE Life Experience Interview: Total Score	*Mixed*	*r* = .164*, p* = .078 NCA: .22/.18	*r* = .001*, p* = .987 NCA: .35/.27	*r* = .048*, p* = .564 NCA: .31/.27
Sense of Mastery	*Non-cognitive*	*r* = .246*, p* = .007 NCA: .11/.06	*r* = -.026*, p* = .752 NCA: .00/.00 ^2^ (.13/.09)	*r* = .066*, p* = .433 NCA: .00/.00 ^2^ (.11/.10)
Openness	*Non-cognitive*	*r* = .089*, p* = .339 NCA: .19/.14	*r* = .030*, p* = .725 NCA: .25/.21	*r* = -.042*, p* = .616 NCA: .22/.16
Reflectivity	*Cognitive*	*r* = .075*, p* = .423 NCA: .16/.11	*r* = -.045*, p* = .588 NCA: .28/.22	*r* = .006*, p* = .947 NCA: .24/.18
Empathy	*Non-cognitive*	*r* = .027*, p* = .774 NCA: .26/.18	*r* = .032*, p* = .698 NCA: .46/.39	*r* = .005*, p* = .957 NCA: .42/.31
Emotion Regulation	*Non-cognitive*	*r* = .091*, p* = .327 NCA: .31/.23	*r* = .030*, p* = .718 NCA: .41/.29	*r* = .145*, p* = .082 NCA: .37/.29
***Self-Report Measures***				
Three-Dimensional Wisdom Scale: Total Score	*Mixed*	*r* = .246*, p* = .007 NCA: .23/.21	*r* = .371*, p* < .001 NCA: .49/.46	*r* = .538*, p* < .001 NCA: .45/.42
Cognitive Dimension	*Cognitive*	*r* = -.039*, p* = .669 NCA: .08/.07	*r* = .116*, p* = .159 NCA: .31/.28	*r* = .320*, p* < .001 NCA: .36/.32
Reflective Dimension	*Cognitive*	*r* = .444*, p* < .001 NCA: .21/.17	*r* = .445*, p* < .001 NCA: .39/.33	*r* = .600*, p* < .001 NCA: .38/.33
Compassionate Dimension	*Non-cognitive*	*r* = .185*, p* = .043 NCA: .12/.13	*r* = .329*, p* < .001 NCA: .39/.32	*r* = .368*, p* < .001 NCA: .33/.31
Adult Self-Transcendence Inventory: Total Score	*Non-cognitive*	*r* = .399*, p* < .001 NCA: .26/.24	*r* = .480*, p* < .001 NCA: .38/.33	*r* = .607*, p* < .001 NCA: .35/.31

^1^ The first number in parentheses is the CE-FDH effect size; the second number is the CR-FDH effect size.

^2^ The zero effect sizes were caused by one outlier who had the highest mean in the sense of mastery subscale and the lowest mean in PWB and life-phase ladder in the sample. The numbers in parentheses are the effect sizes without this case

Table 4 shows considerable divergence between the correlational results and the results of the NCA analyses. Almost all correlations of the open-ended measures with well-being were insignificant, whereas almost all correlations of the self-report measures with well-being were significant. The NCA effect sizes, however, quite consistently indicated medium to large effect sizes for both open-ended and self-report measures.

To quantify this finding, we treated the correlations in Table 4 as data points and dummy-coded type (open-ended = 0, self-report = 1) and content (cognitive = 0, non-cognitive = 1) of measure. We then computed Spearman rank correlations between the dummy variables and the correlations and effect sizes in Table 4 across the 14 subdimensions of the wisdom measures (excluding the BWP, MORE, and 3DWS total scores) × 3 well-being measures, i.e., across a total of 42 coefficients. There was a significant Spearman rank correlation of *r* = -0.761 (*p* < 0.001) between type of measure and size of the correlations, confirming that the correlations were significantly higher for subdimensions of self-report measures than for those of open-ended measures. There was no significant correlation between measure content (cognitive vs. non-cognitive) and the size of the correlations (*r* = 0.128, *p* = 0.420). The NCA effect sizes were neither correlated with type of measure (CE-FDH: *r* = -0.023, *p* = 0.887; CR-FDH: *r* = 0.096, *p* = 0.545) nor with measure content (CE-FDH: *r* = -0.224, *p* = 0.153; CR-FDH: *r* = -0.229, *p* = 0.145). In other words, while the correlations with well-being were far higher for self-report measures of wisdom than for open-ended measures, the NCA effect sizes did not differ between self-report and open-ended measures, and neither the correlations nor the effect sizes were affected by whether the respective wisdom scale or subscale was measuring a cognitive or non-cognitive component of wisdom.

## Discussion

This paper analyzed the relationships between four different measures of wisdom and three different measures of the well-being complex (general life satisfaction, current life phase compared to “best” and “worst” life phase, and psychological well-being), reporting correlations and effect sizes from Necessary Condition Analysis (NCA).

### Most Relationships Between Wisdom and Well-Being Are Triangular

Our first prediction was that relationships between wisdom and well-being would be triangular, especially for open-ended measures of wisdom. That is, highly wise individuals should be high in well-being, whereas individuals low in wisdom would show a wide range of levels of well-being. This prediction was largely supported; most scatterplots in Fig. 2 and the NCA results indicated a triangular pattern. The pattern was particularly clear for the two open-ended measures of wisdom, the Berlin Wisdom Paradigm and the MORE Life Experience Model, where we found a considerable number of participants high in well-being and low in wisdom, but no participants high in wisdom and low in well-being. Notably, most correlations between these two wisdom measures and the well-being variables were zero. As discussed earlier, a likely reason for the discrepancy between correlations and NCA results is the difference in the score distributions between the open-ended wisdom measures and the well-being measures. As Table 3 and Fig. 2 show, few participants scored high in the open-ended measures of wisdom, while many participants scored high in the well-being measures. This difference in distributions limits the size of possible correlations. Substantively, if many people are happy but few people are wise, then even if all wise people are happy, the correlation between happiness and wisdom will be low or zero. This was the pattern that the NCA results confirmed for the open-ended wisdom measures.

The two self-report measures of wisdom, the Adult Self-Transcendence Inventory and the Three-Dimensional Wisdom Scale, had score distributions more similar to those of the well-being measures, with most participants scoring in the upper half of the scale. Accordingly, the relationship with well-being was more linear, as indicated by mostly significant correlations. However, Fig. 2 and the NCA results showed that even for the self-report scales, there were many participants high in well-being and low in wisdom and no participants high in wisdom and low in well-being, at least for life satisfaction and the “life-phase ladder.” The differences between the three well-being measures will be discussed later.

In sum, our findings suggest that even if highly wise individuals are more willing to face the difficult aspects of the human existence than other people, they are still able to maintain high levels of well-being (Ardelt, 2019). These findings do not necessarily contradict the notion that wise individuals may be more willing than other people to consider the darker aspects of human existence (Baltes & Kunzmann, 2003; Staudinger & Glück, 2011). Weststrate and Glück (2017b) argued that there are three reasons why wise individuals may be high in well-being even though they do engage in these thoughts. First, wise individuals are experts at coping with life challenges; their experience has taught them to manage difficult situations and regulate negative emotions. Second, because of their experience with hardship and their awareness of uncertainty, wise individuals may appreciate small pleasures and relish good moments even during difficult times. Third, wise individuals know themselves well and have learned to live their lives in the way that is “right” for them, providing them with resources, such as friends and leisure activities, that can support them in challenging times. With respect to the current study, another relevant aspect is that all three well-being measures assess summative, overall evaluations of one’s life, life phase, or self. Conceivably, individuals could score high in these measures even while they are experiencing a considerable amount of negative affect in their daily life. People working in professions that regularly confront them with suffering or death, for example, may still experience high levels of eudaimonic well-being and life satisfaction. More fine-grained studies of everyday affect in relation to wisdom are needed to test this hypothesis.

We would like to mention one observation that we cannot investigate comprehensively with the present data. We noticed that the participants who scored highest in the open-ended measures of wisdom did not necessarily report the maximum possible values in the well-being measures –in Fig. 2, their dots tend to be slightly below the top of the scale. Our sample of high scorers is too small for an in-depth analysis of this phenomenon, but we believe that individuals high in open-ended measures of wisdom might use the response scales of self-report scales in a somewhat more modest or self-reflective way (Aldwin, 2009; Glück, 2018).

### Relationships with Well-Being Are Similar for Cognitive and Non-Cognitive Wisdom Measures

Our second prediction was that non-cognitive components of wisdom would have stronger relationships with well-being than cognitive components. Unexpectedly, however, content of the wisdom measures seemed to be much less relevant for their relationship with well-being than type of measure. In fact, the BWP, which assesses cognitive aspects of wise thinking about theoretical life problems, showed the exact same pattern of zero correlations and medium to large NCA effect sizes with the three measures of well-being as the MORE interview, which assesses mostly non-cognitive aspects of wisdom from narratives about autobiographical life challenges. We consider it as quite remarkable that even the Berlin Wisdom Paradigm showed a clear triangular relationship with the well-being variables. In other words, all participants who displayed factual and procedural life knowledge, an awareness of the relativity and contextuality of people’s perspectives, and awareness of uncertainty and unpredictability described themselves as quite high in various facets of well-being. We believe that this finding supports the idea of a common core of cognitive and non-cognitive wisdom conceptions: although different conceptions and measures of wisdom tend to emphasize one or the other “side,” actual wisdom may require an integrative interaction of cognitive and non-cognitive components (Baltes & Staudinger, 2000; Glück & Weststrate, in press).

That said, some subdimensions of wisdom did show stronger relationships with well-being than others, but these differences did not always follow the distinction between cognitive and non-cognitive subdimensions. For the BWP, NCA showed that procedural knowledge, value relativism, and contextualism had stronger relationships with well-being than factual knowledge and awareness of uncertainty. In the MORE interview, empathy and emotion regulation had larger NCA effect sizes than openness, reflectivity, and especially sense of mastery. In the 3DWS, the reflective dimension (a willingness to take different perspectives on issues) and the compassionate dimension were more strongly related to well-being, in terms of both correlations and NCA effect sizes, than the cognitive dimension (striving to understand life and learn from experiences). The ASTI, which measures self-transcendence without any subdimensions, was strongly related to well-being.

Thus, as expected, affective components of wisdom such as self-transcendence, compassion, and emotion regulation were quite strongly related to well-being. Unexpectedly, however, equally strong relationships with well-being were found for wisdom components reflecting an awareness of differences in perspectives, values, and contexts (BWP value relativism and contextualism, 3DWS reflective dimension). The weakest relationships with well-being were found for cognitive components referring to awareness of uncertainty and uncontrollability (BWP uncertainty, MORE sense of mastery), complex thinking and self-reflection (MORE reflectivity), and openness and curiosity (MORE openness and 3DWS cognitive dimension).

These differences between subdimensions should not be overinterpreted, especially as NCA results tend to be somewhat susceptible to the presence of outliers (Dul, 2021). Still, it seems highly interesting that well-being is related not only to affective components of wisdom, but also to a general awareness and tolerance of differences between people. Arguably, individuals who consider diversity and individual differences as a source of new insights and ideas are happier and more at peace with their life than people who consider divergent perspectives as a challenge to their own views. For wise individuals, well-being may be closely related to positive and enriching social relationships (Igarashi et al., 2018; Weststrate & Glück, 2017b).

### Wisdom Is Related to Many Aspects of Well-Being

Our third prediction was that the three well-being measures would differ in their relationships with wisdom. We expected the strongest relationship for psychological well-being (Ryff & Keyes, 1995), which represents an eudaimonic conception of a good and meaningful life. We also expected relatively strong relationships between wisdom and general life satisfaction (Pavot et al., 1998), a broad evaluation of one’s past, current, and future life. We expected to find a somewhat weaker relationship between wisdom and our adapted version of the Cantril ladder, where participants rated their current life phase relative to the best and worse phases of their life.

Our results partly supported these predictions. The open-ended measures of wisdom had similar relationships with all three well-being measures. The self-report measures were most strongly related to psychological well-being. For the ASTI, this makes a lot of sense given the considerable conceptual overlap between self-transcendence and psychological well-being, especially with respect to self-acceptance and personal growth (Koller et al., 2017). The dimensions of the 3DWS are somewhat more distant from psychological well-being, but there certainly is some overlap especially with the PWB subdimensions of personal growth, positive relations to others, and autonomy. The relationships of the self-report wisdom measures with the two other well-being measures did not differ much; if anything, the correlations with the life-phase ladder were somewhat higher than those with life satisfaction. In other words, the extent to which participants considered their current life phase as the best was somewhat more strongly related to wisdom than general life satisfaction. One could speculate that highly wise individuals tend to consider their current life phase as their best because are aware of how they have grown and developed over the course of their life.

### NCA: A Promising New Approach for Analyzing a Frequent Type of Relationship

Finally, we would like to add some comments on Necessary Condition Analysis as a novel approach for analyzing nonlinear relationships between psychological variables. In spite of some technical problems that still need to be resolved, such as the influence of outliers (see Dul, 2021), we consider NCA as a highly promising tool for identifying a type of relationships among psychological variables that has long been overlooked. Notably, that type of relationships is not limited to situations where one variable is *theoretically* a necessary condition for the other. The case of well-being and wisdom is a good example: Technically, our NCA analyses tested the hypothesis that high well-being is a necessary (but not sufficient) condition for high wisdom. Theoretically, however, we do not believe that this is the only plausible account of the relationship. It does seem likely that a minimum level of well-being is necessary for people to gain wisdom from experiences; Staudinger and Kunzmann (2005) argued that “a certain level of adjustment is a necessary, but by no means a sufficient condition for growth” (p. 321). Fredrickson’s Broaden and Build theory, for example, suggests that the experience of happiness and joy is necessary for exploring different perspectives and gaining new insights (Fredrickson, 2001). At the same time, high well-being could also be an *outcome* of wisdom—because wisdom enables individuals to see the good things even in bad things, for example, or because wisdom entails effective emotion regulation. Also, high well-being may *co-develop* with wisdom, as some of the factors that foster the development of wisdom may also foster well-being (Weststrate & Glück, 2017b). At the same time, well-being is clearly also attainable through other developmental pathways that result in high well-being without high wisdom (see also Bauer et al., 2019). The cross-sectional data analyzed here do not allow us to distinguish between these different accounts of the relationship between wisdom and well-being. Statistically, however, all of them lead to the same prediction: that high wisdom is associated with high well-being, but low wisdom is not necessarily associated with low well-being. In this way, NCA as a purely descriptive instrument for testing the “triangularity” of relationships could be used to test a broader range of predictions than just those where one variable is theoretically a necessary condition for another.

In sum, the findings of this study indicate that wise individuals are happy, satisfied, and at peace with themselves and their lives. Even as they are willing to look into the darker sides of the human existence and certainly have their darker moments, they know how to live well and they live the life that is right for them. Many individuals who are not particularly wise, however, are equally happy and satisfied with their lives. The complex, dynamic relationship between wisdom and well-being remains an exciting topic for future research.

## Footnotes

1 We are very grateful to an anonymous reviewer of an earlier version of this paper for pointing us to this novel analytical approach!
